# Parenting and the workplace: The construction of parenting protections in United States law

**DOI:** 10.1186/1746-4358-3-14

**Published:** 2008-08-04

**Authors:** Maxine Eichner

**Affiliations:** 1 School of Law, University of North Carolina at Chapel Hill, Chapel Hill, North Carolina, USA

## Abstract

In this paper, I discuss the shortcomings of the legal protections that exist for pregnancy, breastfeeding, and parenting for United States' workers. The two main sources of protection for pregnancy and parenting in United States employment law are the Pregnancy Discrimination Act (PDA) and the Family and Medical Leave Act (FMLA). Both, I argue, contain inadequate protections for the needs of pregnant women and breastfeeding mothers, as well as their infants. I consider what it is about the way these statutes conceptualize the needs of pregnant women, mothers, and their babies, that prevents more robust protection of their needs. I then compare the minimal protection afforded American women and families with more progressive policies in other countries to highlight the possibilities that arise when the state affirmatively supports working parents and their children.

## Debate

In this paper, I discuss the protections that exist for pregnancy, breastfeeding, and parenting for United States' workers and discuss the problems with this set of protections. To cut to the chase, the United States provides a really limited set of protections for pregnancy, breastfeeding and parenting, far more limited than almost every other country (and I really do mean almost *every *other country) for two reasons.

First, U.S. law conceptualizes these issues in a narrow, individual rights/antidiscrimination framework. This framework basically says that women need to be treated the same as men; so long as they are, employers can treat employees of both sexes badly, and fail to accommodate pregnancy, breastfeeding and parenting.

Second, U.S. law is based on a medical model of what counts as a need. If a parent's "need" is medical in the narrowest sense of word s/he might get protection. So employees get protection for leave for complications of pregnancy, birth, and serious medical conditions of their children. If a parent's need is not medical in this narrow sense, it is not covered by law. The law deems activities like breastfeeding to be a "personal choice," rather than a need. And, in so doing, it says that society does not have a responsibility to support that choice. It does not matter if there are good reasons connected to children's and society's welfare; contrary to these needs, the law provides no protection.

I will briefly describe the two main sources of protection for pregnancy and parenting in United States employment law: the Pregnancy Discrimination Act (PDA) and the Family and Medical Leave Act (FMLA) [1,2]. I then compare the minimal protection afforded to American women and families with more progressive policies in other countries to highlight the possibilities that arise when the state affirmatively supports working parents and their children.

### The pregnancy discrimination act

The first major source of protection for parenting in the United States comes from Title VII of the Civil Rights Act of 1964, the federal statute that prohibits workplace discrimination based on sex [3]. It is bizarre that one of the main sources of protection for parenting in U.S. law is a sex discrimination statute, given all the political rhetoric in the U.S. about how important families are and how ambivalent the nation is about sex equality, but that is how U.S. law has developed. Basically, Title VII requires equal treatment of men and women. When the statute was first enacted, the United States Supreme Court held that discriminating based on pregnancy did not constitute discrimination based on sex; according to the Court, pregnancy discrimination involved discriminating against "pregnant persons" rather than women, ignoring the obvious fact that only women have the potential to become pregnant [4]. In response, Congress amended Title VII in 1978 to add the Pregnancy Discrimination Act, declaring that discrimination based on pregnancy constituted prohibited sex discrimination [1].

Title VII and the PDA have indisputably had many positive effects on the lives of working women in America. For example, they ended the once common employment practice of firing all women in particular jobs who were pregnant. What they do not do, however, is ensure any positive level of treatment for pregnancy. Pregnancy can be treated as well or as poorly as any other health issue that causes people to miss work. If an employer has a sick leave policy, then it has to include pregnant employees in that policy. But the PDA does not require employers to have a sick leave policy in place at all. By the same token, Title VII requires women be treated the same as men, but it does not require that either sex be treated well, or that employers support or even accommodate them in bearing and rearing healthy children.

In addition, courts have interpreted the PDA to deny breastfeeding protection [5]. Rather than consider it a "related medical condition" with respect to pregnancy, which would give it coverage under the PDA, courts deem breastfeeding to be a "choice" related to parenting and therefore to be uncovered [6]. Constructing breastfeeding as a choice that absolves employers from any duty to accommodate it evades the question of whether such "choices," when they contribute to welfare of children, should be supported. It also makes it clear that employees are protected only for disabilities; and breastfeeding does not fit within this disability model.

### The family and medical leave act of 1993

The FMLA is the sole exception to the narrow, nondiscrimination framework provided for pregnancy protection in the U.S. In contrast to Title VII, the FMLA seeks to provide a substantive floor for treatment of pregnancy and other health conditions. It gives employees up to twelve weeks of unpaid leave for pregnancy, childbirth, and care of ill children [7].

There are many limits to the protection offered by the FMLA, however. First, the statute only applies to employers with 50 or more employees; this means that only 5% of employers are covered [8]. In addition, since the FMLA only grants the right to unpaid leave, the majority of covered employees do not take their granted leave because they cannot afford to be out of work [9]. Furthermore, like the PDA, the FMLA is largely built on a medical model. It covers only serious health conditions, and the needs of newborns [9]. It does not take into account that caring for children takes far more than twelve weeks, and that children need their parents for far more than just serious medical emergencies. Because of this, breastfeeding and much else of parenting, is not covered by the FMLA.

### An international perspective

It is sobering to compare the package of benefits for pregnant and working mothers in the U.S. with what the rest of the world has offered these parents for decades. A recent Harvard study found that workplace protections for families in the U.S. are weaker than those of all high-income countries and most middle and low income countries [10]. The study found that, in contrast to the U.S., 163 countries guaranteed paid maternity or parental leave to new mothers and 45 ensure that the father receives paid paternity or parental leave [10]. Notably, the study found that the U.S. is one of only five countries out of 173 in the survey that did not guarantee some form of paid maternity leave; the others are Lesotho, Liberia, Swaziland and Papua New Guinea [11].

The U.S. also lags far behind *all *other industrialized countries in enabling workers to care for sick children and other family members [10]. At least 145 countries provide paid sick days, with 127 providing a week or more annually [10]. In contrast, the U.S. provides unpaid leave through the FMLA, and there is no federal law providing for paid sick days.

In contrast to the U.S., with its reliance on an antidiscrimination model that provides only the relatively shallow guarantee of formal equality, other countries' protections frame family interests in a manner that promotes more robust protection. For example, Western European nations that offer far better protections than ours, do so by providing substantive guarantees of sex equality, and by harmonizing this interest with other interests, such as the health of children, workers' families, and the soundness of community. These countries also frame families' and children's protections in a manner that moves beyond a medical model. They start from the premise that child bearing and child rearing are societal goods, and their laws reflect a substantial commitment to enabling women and men to work and have a family. On this basis, their laws mandate employer accommodation and support for pregnancy and childbirth. By following the lead of these countries in providing substantive protections, the United States could provide far better support to working parents and their families.

## Competing interests

The author declares that she has no competing interests.
